# Commentary: Evaluation of Models of Parkinson's Disease

**DOI:** 10.3389/fnins.2016.00283

**Published:** 2016-06-21

**Authors:** Pollyana C. Leal, Lívia C. R. F. Lins, Auderlan M. de Gois, Murilo Marchioro, José R. Santos

**Affiliations:** ^1^Department of Physiology, Federal University of SergipeSão Cristóvão, Brazil; ^2^Behavioral and Evolutionary Neurobiology Laboratory, Department of Biosciences, Federal University of SergipeItabaiana, Brazil

**Keywords:** MPTP, 6-OHDA, reserpine, serotonin, neurodegeneration, Parkinson disease

In the recently published review article by Jagmag et al. (2016), some currently available neurotoxin based and genetic models of Parkinson's disease (PD) were described, highlighting the advantages and disadvantages of preclinical models use to knowledge of PD. The aim of this commentary is to shed light and to make a reflection on some fundamental issues involving the pathophysiology of PD and additionally bring to the discussion the animal model of PD based on the administration of reserpine (RES).

The pathological hallmark of PD involves the progressive loss of neurons in the substantia nigra pars compacta (SNpc) (Politis and Niccolini, 2015). However, a large body of evidences implies that the PD pathology is a multisystemic degenerative process that involves other neurotransmitters such as serotonin (5-HT) and norepinephrine (NE) (Deusser et al., 2015; Liu et al., 2015; Politis and Niccolini, 2015). It has been demonstrated that the serotonergic system may be the most critical neurochemical system involved in the pathology of PD, after the dopaminergic system (Huot and Fox, 2013; Liguori et al., 2015). Changes in serotonin levels may be a contributing factor to PD symptomatology, in particular, non-motor disturbances (Loane et al., 2013). Patients with PD show loss of serotonergic neurons in the brainstem raphe nuclei (Braak et al., 2003) and reduced expression of tryptophan hydroxylase type 2 (TPH2) in the median raphe nucleus (MnR) (Kovacs et al., 2003). According to Braak staging of PD pathology, serotonergic cell loss in the raphe nuclei is evident prior to nigrostriatal dopaminergic degeneration. Interestingly, the pattern of serotonergic loss also seems to be different from that observed in the dopaminergic system (Politis and Loane, 2011). Additionally, other neuronal systems, including noradrenergic locus coeruleus, are also affected in PD (see review in Jellinger, 1999) and they have been linked to non-motor symptoms of PD as well. Thereby, these evidences of alterations in the raphe nuclei and catecholaminergic nuclei highlight the importance of looking beyond the nigrostriatal system in the PD study, in order to elucidate the underlying mechanisms of deficits of other neurotransmitter systems in the physiopathology of PD and provide useful information for the development of therapeutic strategies for this disease.

In the paper, Jagmag et al. (2016) highlight toxins widely used as animal models of PD. The toxin 1-methyl-4-phenylpyridinium (MPP+), active metabolite of MPTP, is taken up into dopaminergic terminals by the dopamine transporter (DAT) showing the high affinity for dopaminergic vesicles (Tipton and Singer, 1993; Dauer and Przedborski, 2003). According Andrew et al. (1993), 6-hydroxidopamine (6-OHDA) is elevated in the urine and striatum of L-DOPA treated PD patients, suggesting that 6-OHDA may be an endogenous neurotoxin. This toxin is taken up into dopaminergic terminals by DAT as well as taken up to noradrenergic terminals by the noradrenergic transporter (NAT). However, a lot of studies using 6-OHDA model given an inhibitor of NAT as a pretreatment, in order to protect noradrenergic terminal from 6-OHDA toxicity, precluding the possibility of PD symptomatology analysis as observed in PD patients. Pesticide-induced model, particularly rotenone and paraquat, have substantial challenges of replicability due the high mortality observed in rats. In addition, these pesticides induce selective degeneration of dopaminergic neurons as illustrated in Figure 1.

**Figure 1:**
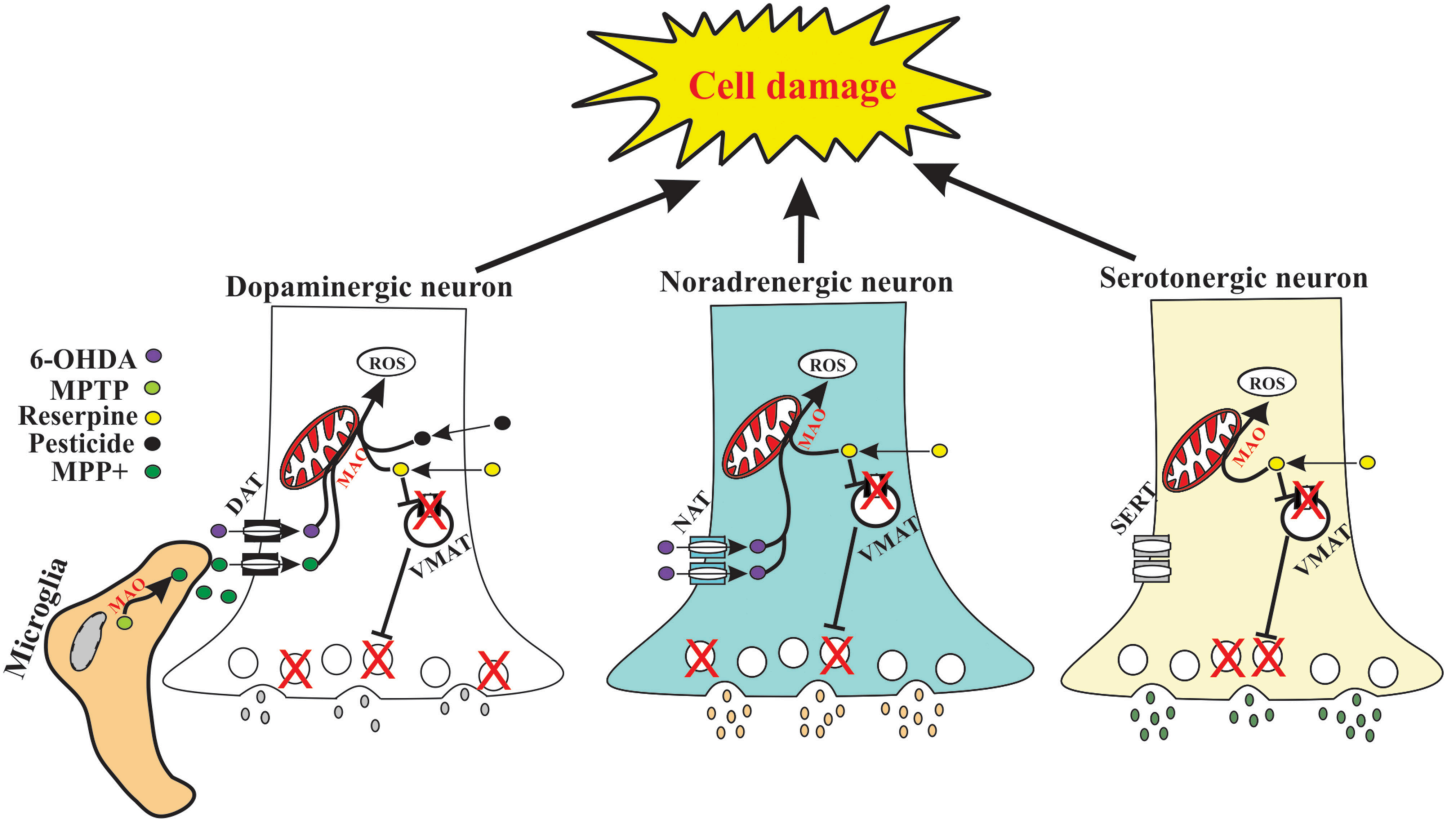
**Schematic representation of molecular events in the dopaminergic (left), noradrenergic (center) and serotoninergic (right) neurons after administration of the main toxins (MPTP, 6-OHDA, Pesticide and Reserpine) used to induce animal models of PD**. The 1-methyl-4-phenyl-1,2,3,6-tetrahydropyridine (MPTP) toxin is converted by microglia to 1-methyl-4-phenylpyridinium (MPP+), which is taken up via dopamine transporter (DAT) by dopaminergic neurons. Once inside neuron, MPP+ acts via inhibition of complex-I of the respiratory chain. Pesticides (Rotenone and Paraquat) are also captured by DAT and have direct action on the complex-I of the respiratory chain. The 6-Hydroxydopamine (6-OHDA) is a neurotoxin that acts equally on dopaminergic and noradrenergic neurons, entering in these neurons via DAT and noradrenergic transporter (NAT), respectively. The Reserpine inhibits the vesicular monoamine transporters (VMAT), inducing a loss of storage capacity of monoamines in synaptic vesicles, monoamines depletion in nerve terminals and in an abnormal cytosolic accumulation of monoamines. The monoamine oxidase (MAO) catalyze the oxidation of monoamines. Thus, the Reserpine acts on dopaminergic, noradrenergic and serotonergic neurons. The toxins mentioned above increase the production of reactive oxygen species (ROS), which leads to oxidative stress and cell damage via different routes.

Jagmag and colleagues did not regard the animal model of PD induced by Reserpine (RES). Reserpine model was one of the first models to investigate the pathophysiology and to demonstrate the therapeutic efficacy of L-DOPA, which remains the gold-standard treatment for PD (Carlsson et al., 1957). However, in the 1980s, this toxin became underused due the lack of selectivity for dopamine was considered a failure of the model. The RES is an ester alkaloid derived from *Rauwolfia species* root that induces symptoms, neurochemical and pharmacological alterations in humans (May and Voegele, 1956) and animals (Fernandes et al., 2012; Santos et al., 2013; Leão et al., 2015) similar to those seen in PD patients. It is an inhibitor of vesicular monoamine transporters (VMAT) as presented in Figure 1. These transporters play an essential role in the presynaptic neurotransmission control and in the regulation of cytoplasmic levels of monoamines. The inhibition of VMAT by reserpine results in a loss of storage capacity of monoamines in synaptic vesicles, causing depletion of brain and peripheral monoamines (Dopamine, NE and 5-HT) leading to oxidative stress (Vergo et al., 2007). Depletion of monoamines, especially of dopamine, and oxidative stress are important features of pathophysiology of PD (Miller and O'Callaghan, 2015). Reserpine is also able to induce motor and non-motor deficits in rodents, such as oral dyskinesia, muscle rigidity (Colpaert, 1987; Neisewander et al., 1994; Fernandes et al., 2012), recognition memory deficits (Santos et al., 2013; Sarmento et al., 2015), anxiety and depressive-like behavior (Santos et al., 2013; Antkiewicz-Michaluk et al., 2014), sleep abnormalities (Chen and Marsh, 2014) and gastrointestinal dysfunction such as gastric dysmotility and constipation (Pellegrini et al., 2015). Thus, the reserpine model is a good mimic of the disease biochemistry and an important model of choice for early preclinical stages of drug discovery programmes.

Our research group has proposed that repeated administration of low doses of reserpine can mimic the progressive nature of PD (Santos et al., 2013). In this model, the animals showed cognitive and emotional deficits in the early stages, even before the onset of motor abnormalities. The non-motor symptoms have been associated mainly to impairments in the serotonergic and noradrenergic pathways. The reserpine model has advantages and disadvantages like others animal models of PD. The main advantage of this model is its ability to produce symptoms similar to those observed in the early stages of PD, may be due to monoamines loss (Dopamine, NE and 5-HT). However, the underlying mechanisms of reserpine toxic effects on monoaminergic neurons are not completely understood. We believe that no single PD model is currently available; on the other hand, there are distinct models that allow us to investigate specific mechanisms of PD, since different mechanisms lead to neuronal cell death in PD and parkinsonian patients exhibit heterogeneous non-motor and motor symptoms.

## Author contributions

All authors participated in the preparation and discussion of the commentary. Designed and organized the illustration: AG.

### Conflict of interest statement

The authors declare that the research was conducted in the absence of any commercial or financial relationships that could be construed as a potential conflict of interest.
